# Perforation of the Nasal Septum

**Published:** 1915-05

**Authors:** John C. Warbrick

**Affiliations:** Chicago


### Perforation of the Nasal Septum

By JOHN C. WARBRICK, M. D., Chicago.

The bones forming the nasal septum or partition are the following: crest of the nasal spine, nasal spine of the frontal bone, perpendicular plate of the ethmoid, the vomer, part of rostrum of sphenoid and the crests of the maxillary and palate bones.

While perforation of the septum is not very common and does not occur nearly so often as deviation still a good many cases are to be found both in men and women.

The perforation may take place through either the cartilaginous or bony part of the septum and the size of the opening may vary from that of a pin head in the beginning to almost complete destruction of the whole structure back to the post nasal space leaving only a small part or bridge in front along with the roof of the nose to serve as a means of protection.

Thus the nasal passages are made into one large cavity extending from the front of the nose to the back while in some cases both the middle and inferior turbinated bodies may be entirely destroyed thus making the cavity much larger. Sometimes in cases such as these the bridge of the nose may cave in a good deal causing a depression the shape of a saddle to form. This is called the saddle back nose on account of its resemblance to the saddle of a horse. The condition may be brought about in some cases by syphilis, scrofula or chronic catarrhal states in some individuals lasting for years. To perforate the cartilaginous part of the septum would not seem to be a very difficult thing to do when we take into consideration its fragile nature and thinness, especially if it become weakened in any way either by local or general conditions.

In the former case a deviation may be mentioned and also a constant bleeding may occur from time to time at some point on the anterior part of it which may tend to weaken it some.

In the latter instance some constitutional conditions such as disease of the heart, of the fatty type, which impairs the circulation may tend to have some effect upon it, also chronic catarrhal states in some persons predisposed that way.

The causes of septal perforation may be local and general; traumatic or idiopathic. Under the head of traumatism come blows received on the nose in various ways as from a bat or fist in boxing.

The use of surgical instruments internally in removing spurs or doing other operations may cause a perforation.

Other causes are the use of caustics, septal deviation, abscesses, lupus, typhoid fever, diphtheria, syphilis, scrofula, dryness of the nose, tuberculosis.

Sometimes the constant picking at the septum with the finger nail to remove a scab or other material may in time cause ulceration and lead to perforation.

Perforation from irritation of any kind may begin as a small, clean cut, ulcer in the cartilaginous part. This gradually erodes the thin layer of tissue over the cartilage and in time penetrates it by making a small opening, the tendency for which is to increase in size by degrees instead of healing over. The shape of the perforation may be round or oval both through the tissue and cartilage with edges even at first, but as the opening gets larger there may be some recession of the tissue in one or both nostrils exposing the cartilage, due possibly to irritation of some kind causing ulceration of the edges along with occasional bleeding.

Sometimes blowing the nose hard to free the passages of crusts, mucous or other irritating particles may cause further ulceration and hemorrhage.

Occasionally a wall of thickened tissue circular in shape may form behind a perforation and serve as a barrier to prevent it extending backwards very quickly if it has a tendency to do so.

An opening through the bony part of the septum may in many cases be due to syphilis or scrofula, and more so the former when part or the whole septum may be destroyed along with some of the membrane linings, the floor of the nose giving it a foul looking appearance: The edges of the opening may be irregular and ragged, also ulcerated with a tendency to bleed when touched.

When a perforation once occurs it may remain stationary for a time in size but there is not much chance of healing the opening over unless at the very first when conditions might be favorable in some cases.

The tendency would seem to be for the opening to increase in size and extend by degrees and more so when due to syphilis for unless checked by some means syphilitic ulcers tend to increase and spread rapidly. In such cases it may extend forwards, upwards and backwards by the erosion and necrosis of the tissues and gradual destruction of the cartilage and bone.

To have a perforation either large or small through any part of the septum is somewhat annoying and trying especially to some temperaments that are easily irritated and this condition tends to make them so.

Crusts and scabs may form about the edges of the opening and become firmly adherent so that they can only be removed with difficulty and when they are bleeding may readily occur.

When the nose is blown hard the crusts and scabs in some cases may pass from one nostril into the other, and more so in patients with narrow passages, thus causing a good deal of bother and making it more difficult to expel them.

The formation of crusts and scabs about the edges of a perforation may cause a good deal of itching and tickling from time to time especially when the opening is in the front part of the septum. This causes a good deal of irritation to the patient and tends to excite frequent blowing of the nostrils to expel them if possible from time to time which may not be easily done. In some cases however most of the septum may be destroyed without causing very much inconvenience to the patient either from breathing or from the presence of crusts which in some of these cases do not seem to form so readily as when the opening is smaller. When there is a large opening the crusts and scabs have no place to form except above and on the sides of the nostrils, so they are easily detached and removed from the passages when they form, so cause no trouble in the front of the passages. Just what is the commonest cause of septal perforation is not easy to state but possibly syphilis may be the chief offender in a good many of the cases or cause it more than anything else.

Few cases of perforation it seems to me can be classed as idiopathic, no apparent cause being noted for there must always be some agent to act either locally or on the general system to produce it although it might not be found.

The treatment of a septal perforation of a small or a large size is not very satisfactory so far as restoration of the eroded part is concerned either in the cartilaginous or bony part, for it seldom if ever occurs from treatment of any kind given and more so when due to syphilis.

In some cases when the opening is small and stationary in a healthy person in the cartilage there might be a chance to fill it in if from an ordinary cause but even then everything being favorable it would require a good deal of time and patience and be difficult to do on account of mucous and crusts forming in the passages causing irritation along with other foreign material getting into the nostrils to cause ulceration.

To restore a larger perforation in any part of the septum is almost an impossibility. When the opening is small and in the front part of the nose it is about the best time to try and close it if possible.

Silver nitrate may be used to heal the edges and help promote the growth of the tissue surrounding the opening.

A flap operation of some kind may be done if conditions are favorable in the nostrils to close over the space.

A small piece of thin bone may be inserted if possible on one side between the cartilage and tissue and nitrate of silver used to heal the surface over by promoting the growth of the tissue. The same method may be tried with a small piece of ivory. In a small perforation that cannot be closed or in fact any kind large or small the object to be attained would seem to be to keep it from getting larger. This can be done by keeping the nasal passages as free as possible of mucous and crusts by spraying it out. The edges should be kept clean and any ulcerations healed over by using silver nitrate whenever necessary to prevent bleeding.

Where there is a large perforation in the septum due to syphilis with necrosed tissue on the floor of the nose with a tendency to bleed the following method is useful to use. Cleanse the nostrils by spraying it out and removing all the crusts and scabs, then use Phenol a 5 per cent. solution to apply to all the surfaces. Iodol a 5 per cent. solution may be used in place of phenol or they may be used together. Silver nitrate may then be used twenty or thirty grains to the ounce and well applied to all the surfaces.

---

**X-Rays in the French Army.** The Archives of Medical Electricity, (resumed with Feb. issue after suspension for 5 months due to mobilization of most of the staff) states that fixed installations exist, at Val-de-Grace, Lille, Champs de Chalons, Bourbonne, Epinal, Verdun, Belfort, Lyon, etc. Ten mobiles installations are located at main depots for wounded. In addition, there are two types of cars fitted with apparatus and generator.

Electro-magnetic extraction of missiles (ibid.) Fragment of Howitzer shell, lodged dangerously near crural vessels; healing of wound as first dressing had been made without facilities for X-ray and operative procedures. The instrument used weighed 40 kilos, the magnetic core being 6 c.m. in diameter, the coil using 3.5 amperes at 110 volts. 10-15 minute seances were given daily, closing and opening the circuit. No apparent results were obtained for the first five days. At the eighth seance, the skin was raised 1.5 c.m., the swelling being 10 c.m. long and 6 c.m. wide. A 4 c.m. incision was then made in the skin and the vastus internus divided at a depth of 2.5 c.m. whereupon the foreign body was readily removed. In another case, the foreign body lodged above the elbow and, owing to oedema, could not be readily found by operation. After 18 seances, it was brought into radioscopic view, the traction movement being visible. It was then removed by incision and found to be a fragment of the jacket of a German bullet, much distorted. In a third case, the missile had broken the lower end of the humerus. After 22 seances, the movement of the foreign body, seen by the fluoroscope, was considerable as the current was made and broken. A mass of iron weighing 6 grams was removed.

